# Towards determining perceived audience intent for multimodal social media posts using the theory of reasoned action

**DOI:** 10.1038/s41598-024-60299-w

**Published:** 2024-05-08

**Authors:** Trisha Mittal, Sanjoy Chowdhury, Pooja Guhan, Snikitha Chelluri, Dinesh Manocha

**Affiliations:** 1 Department of Computer Science, University of Maryland, College Park, USA; 2 Department of Electrical and Computer Engineering, University of Maryland, College Park, USA

**Keywords:** Computer science, Information technology

## Abstract

Increasing use of social media has resulted in many detrimental effects in youth. With very little control over multimodal content consumed on these platforms and the false narratives conveyed by these multimodal social media postings, such platforms often impact the mental well-being of the users. To reduce these negative effects of multimodal social media content, an important step is to understand creators’ intent behind sharing content and to educate their social network of this intent. Towards this goal, we propose Intent-o-meter, a perceived human intent prediction model for multimodal (image and text) social media posts. Intent-o-meter models ideas from psychology and cognitive modeling literature, in addition to using the visual and textual features for an improved perceived intent prediction model. Intent-o-meter leverages Theory of Reasoned Action (TRA) factoring in (i) the creator’s attitude towards sharing a post, and (ii) the social norm or perception towards the multimodal post in determining the creator’s intention. We also introduce Intentgram, a dataset of 55K social media posts scraped from public Instagram profiles. We compare Intent-o-meter with state-of-the-art intent prediction approaches on four perceived intent prediction datasets, Intentonomy, MDID, MET-Meme, and Intentgram. We observe that leveraging TRA in addition to visual and textual features—as opposed to using only the latter–results in improved prediction accuracy by up to $7.5\%$ in Top-1 accuracy and $8\%$ in AUC on Intentgram. In summary, we also develop a web browser application mimicking a popular social media platform and show users social media content overlaid with these intent labels. From our analysis, around $70\%$ users confirmed that tagging posts with intent labels helped them become more aware of the content consumed, and they would be open to experimenting with filtering content based on these labels. However, more extensive user evaluation is required to understand how adding such perceived intent labels mitigate the negative effects of social media.

## Introduction

Social media platforms have become an important part of people’s daily lives. Recent surveys^1,2^ show that, compared to 10 years ago, the number of Americans using social media to connect with others, engage with news content, share information, and entertain themselves has increased from 40% to 75%.

The positive influence of social media notwithstanding, more recently, various findings^3^ have brought to light incidents where users are adversely affected by these social media platforms driven by a lack of control over multimodal content consumed by users. A growing trend shows that content posted by a user represents a false narrative designed to uplift the user’s “social status” in society. In other words, users often tend to change their behavior on social media to deliver a positive impression of themselves^4–8^. While, this understanding exists, it is not always reinforced in the minds of the audience who is the recipient of this content. Such content, when consumed by others, leads to issues related to body image, anxiety, and mental health–specifically in teenagers—because of unnecessary negative social comparison^9,10^.

Over the past few years, research has shown that emotions elicited when a user shares content can be transferred over social media networks leading to users experiencing similar emotions^11–13^. This has been shown to be more prevalent in image/video based applications^14,15^. Therefore, to reduce the negative effects of such content shared on social media, an important step is to understand creators’ intent behind sharing content and to educate their social network of this intent to minimize any negative implications^16^. Towards this goal, several efforts have been made to understand this intent behind sharing multimodal social media content^17–21^.

### Perceived human intent

While prior work in this space does not make this distinction, we wish to make it clear to the reader that we are interested in the *perceived creator intent* for a multimodal social media post, more specifically we are focused on audience-perceived creators’ intent. This is important because we do not have groundtruth from creators themselves regarding their intent behind every multimodal post. Furthermore, for the scope of this work, because our goal is to protect social media users from vulnerable multimodal content on social media, it makes more sense to pursue the perceived creator intent. Furthermore, once a message has been created and has left the creator, it is up to the audience to interpret the post, which is another reason why we focus our attention on audience-perceived intent for the creators’ content.

However, understanding this perceived human intent behind such multimodal content is challenging for several reasons. First, there is no standard intent taxonomy that exists specifically to these social media multimodal data. Some of the common taxonomies for perceived intent for social media content have been proposed by Jia et al.^17^, Kruk et al.^18^, Zhang et al.^22^ and, Xu et al.^23^. These prior works scrape posts from various social media platforms like Instagram, Unsplash (https://unsplash.com), Twitter, Weibo, Facebook, and Google Images. However, the intent prediction models proposed by these prior works for such multimodal data are limited to the standard visual and textual understanding. Furthermore, these methods employ black box neural networks that lack explainability and are, in general, susceptible to domain shift issues. With respect to the intent taxonomies, there is a diverse and wide-ranging taxonomy. We have listed these various taxonomies in Suppl Appendix 1, Suppl Table 3. All others seem to be Furthermore, understanding creator intent goes beyond the standard visual recognition tasks and is a psychological task inherent to human cognition and behavior^24–28^.

### Main contributions

The following are the novel contributions of our work.

1. Detecting perceived intent for social media content: we propose Intent-o-meter, a perceived human intent prediction model for multimodal social media posts. In addition to visual (image) and textual (caption) features, Intent-o-meter is modeled on the Theory of Reasoned Action (TRA) by designing new input features for modeling (i) the creator’s attitude towards sharing a post, and (ii) the social norm or perception towards the post in determining the creator’s intention.
2. Educating audience with creator’s intent: we developed a web application, similar to a social media platform, with these predicted intent labels displayed on posts to gather users’ feedback. We tested this application with 100 participants and gathered feedback on the use of such intent labels and its potential impact on reducing the negative effects of social media content on audience.
3. A multimodal social media content intent prediction dataset: we introduce Intentgram, a perceived intent prediction dataset curated from public Instagram profiles using Apify (https://apify.com). At 55K samples consisting of images, captions, and hashtags, with a 7-label intent taxonomy derived from Kruk et al.^18^, Intentgram is the largest ($4\times$ the second largest) dataset to date.

Empirical evaluations on the Intentonomy, MDID, and MET-Meme datasets show that leveraging TRA in addition to visual and textual features results in improved prediction accuracy by up to $7.5\%$ in top-1 accuracy and $8\%$ in AUC on Intentgram. To our knowledge, our perceived intent prediction model is the first to leverage such a theory, modeling attitudes and social norms, in the context of social media. We believe that doing so makes the model take into account social media characteristics and user behavior; and hence results in increased model performance. We also analyzed user feedback on the web application that displayed intent labels alongside posts, and observed that that 70% of users found the intent labels useful.

**Figure 1:**
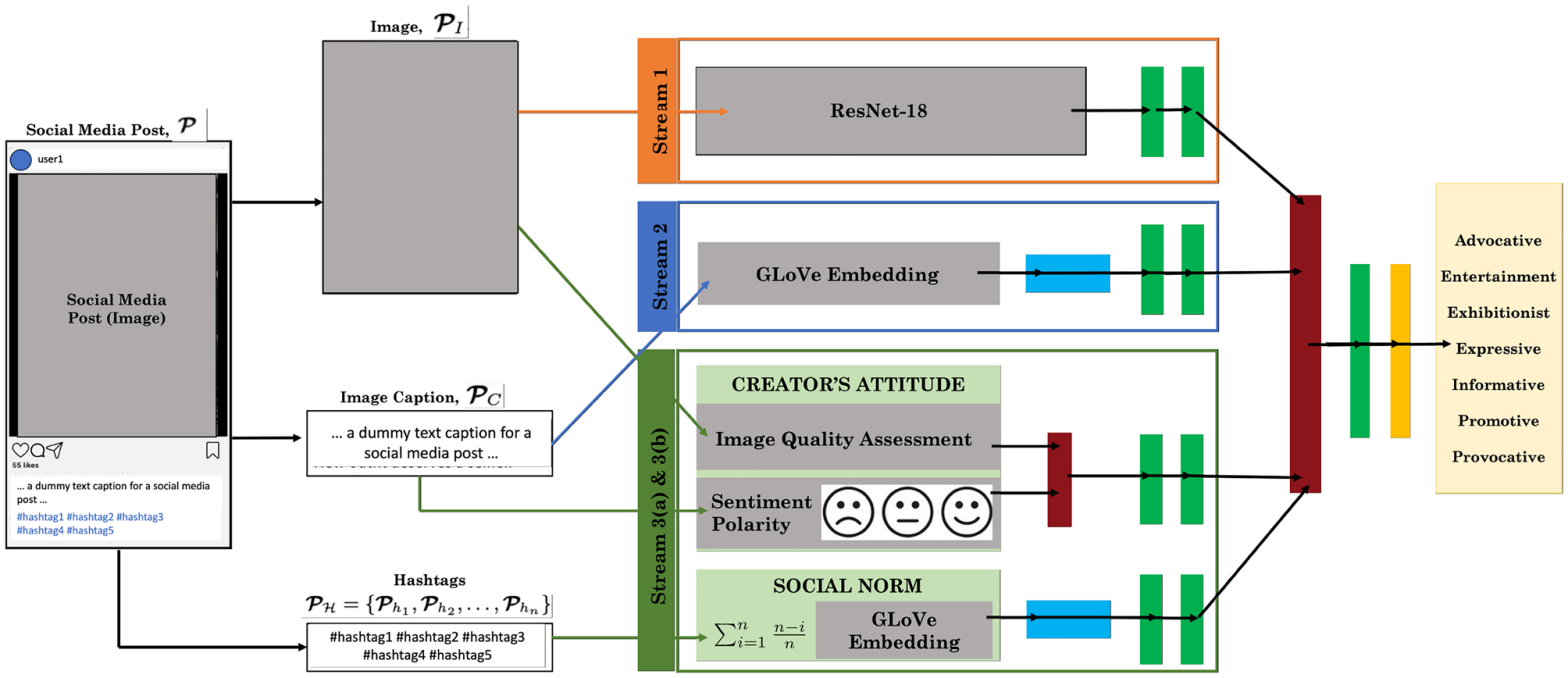
Intent-o-meter: given as input a social media post, $\varvec {{\mathcal{P}}} = \{\varvec {{\mathcal{P}}}_I, \varvec {{\mathcal{P}}}_C, \varvec {{\mathcal{P}}}_{{\mathcal {H}}}\}$, which has three components (an image, $\varvec {{\mathcal{P}}}_I$, with an associated caption, $\varvec {{\mathcal{P}}}_C$, and a set of hashtags, $\varvec {{\mathcal{P}}}_{{\mathcal {H}}} = \{ \varvec {{\mathcal{P}}}_{h_1},\varvec {{\mathcal{P}}}_{h_2}, \dots , \varvec {{\mathcal{P}}}_{h_n}\}$), our goal is to predict the *perceived intent label* for $\varvec {{\mathcal{P}}}$. Intent-o-meter has three streams. In the first stream (orange), we encode the visual features of the image, in the second stream (blue) we encode the captions, and finally, in the third stream (green) we model the Theory of Reasoned Action; both *attitude of the author/creator* and the *social norm of the kind of post*, ${\textbf{P}}$. We then fuse the three streams (dark red) to make the final perceived intent prediction. The networks consist of fully-connected layers (light green), LSTM layer (blue), concatenation operation (dark red), and softmax layer (yellow).

## Related work

In this section, we discuss previous works in related domains. To begin, we first go over the impact that social media can have on mental well-being of users (“Social media’s impact on mental well-being”). We elaborate on the need to infer the intent of social media content in “Measuring perceived intent on social media”. Then in “Social media intent recognition models”, we summarize various datasets and models that have been proposed in the recent past for inferring intent for social media content. We also provide an understanding of the Theory of Reasoned Action and our motivation for using this for our model in “Social media and theory of reasoned action”.

### Social media’s impact on mental well-being

Social media sites like Instagram, Facebook, and Twitter have become an important part of our daily lives, especially for young adults^29,30^. The pressure to publish “socially acceptable” and “socially likable” content often results in a depiction of a false narrative on social media; more specifically image/video-based platforms like Instagram. Sophisticated editing tools and filters add to this false narrative. The impact of such content on young people is of grave concern. They often compare themselves to others (what they see) to assess their opinions and abilities, and such comparison has been known to lead to depression^31^. Such comparisons can have serious impact on physical and mental well-being. Young people also quantify their social acceptance in terms of a number of likes/comments/shares/follows^32^ which again traps them in a vicious circle.

### Measuring perceived intent on social media

“Intent” is a broad term and can be used in various contexts (next steps/plan of agent^33,34^, actions^35^, causal reasons try to identify actions like “play”, “clean”, and “fall” among many others and try to analyze the causal reason behind these actions, emotions, and, attitudes^23^).

However, such interpretations are not enough to answer the question, “Why do people post content on social media platforms?”. A few prior works^17,18,21,22,36^ have proposed datasets and intent taxonomies that can answer the above question. However, there is little consensus among the taxonomies proposed. The pressure to publish “socially likable” content often results in a depiction of a false narrative on social media. Sophisticated editing tools and filters add to this false narrative. The impact of such content on young people is of grave concern, leading to comparing themselves to others (what they see) to assess their opinions and abilities, quantify their social acceptance in terms of number of likes/comments/shares/follows^31,32^. A step towards this is educating and making young adults aware of what to expect on such platforms (intent of the content creator), and ensuring they feel less affected and less vulnerable to what they see.

### Social media intent recognition models

Intent Classification for social media data provides various challenges. As discussed in “Measuring perceived intent on social media” , there is little consensus in existing intent taxonomies built for social media content. We summarize the various datasets and taxonomies for intent prediction for social media data in Suppl Table 3. Some recent works have also explored intent recognition models for various datasets. Kruk et al.^18^ and Zhang et al.^22^ use both visual (image) and textual (captions) modalities to predict an author’s intent for their Instagram posts. Jia et. al.^17^ focus more on predicting intent labels based on the amount of object/context information, and use hashtags as an auxiliary modality to help with better intent prediction. The scope of these works is limited to just the visual and textual features of the data. Understanding human intent, however, is a psychological task^37^, extending beyond standard visual recognition. Therefore, we conjecture that additional cues from social media psychology literature are needed to improve the state-of-the-art in intent prediction.

### Social media and theory of reasoned action

The Theory of Reasoned Action (TRA)^38^ assumes that people make rational choices when they engage in a specific behavior (e.g. *posting a content on social media*), and that behavior is driven by *intentions*. Furthermore, TRA lays out the following two factors that determine *intention*: (i) attitude toward the behavior and (ii) the subjective norms associated with the behavior. Attitudes toward the behavior refers to the overall evaluations of the performance of a behavior in question, and subjective norms refers to perceived pressure or opinion from relevant social networks. Generally, individuals who have more favorable attitudes and perceive stronger subjective norms regarding a behavior are more likely to show greater intentions to perform a behavior. Prior research^39–41^ has used TRA to reason and develop an understanding of what motivates social media users to share information online. They confirm that TRA can be used as a model for social networking behavior. They also find that both intention and subjective norm are positively associated with intention to use social media^42,43^. While these studies, however, confirm TRA and its role in modeling user intent on social media, no work so far uses TRA to *predict* user intent

## Methods

In this section, we present Intent-o-meter, our algorithm for inferring the perceived creator’s intent in social media posts. We formally state the problem and give an overview of our approach. Following that, we explain all the components of our model, Intent-o-meter, in “Intent-o-meter: approach”  to “Fusion: inferring the perceived intent label”.

### Problem statement

#### Problem 1

*Perceived human intent prediction: given as input a social media post*, $\varvec {{\mathcal{P}}} = \{\varvec {{\mathcal{P}}}_I, \varvec {{\mathcal{P}}}_C, \varvec {{\mathcal{P}}}_{{\mathcal {H}}}\}$,* which has three components: an image*, $\varvec {{\mathcal{P}}}_I$,* with an associated caption*, $\varvec {{\mathcal{P}}}_C$,* and a set of hashtags*, $\varvec {{\mathcal{P}}}_{{\mathcal {H}}} = \{ \varvec {{\mathcal{P}}}_{h_1},\varvec {{\mathcal{P}}}_{h_2}, \dots , \varvec {{\mathcal{P}}}_{h_n}\}$,* our goal is to predict the* *perceived intent label for* $\varvec {{\mathcal{P}}}$.

We present an overview of our perceived intent prediction model, Intent-o-meter, in Fig. 1. As our input is multimodal, we refer to multimodal deep learning literature and extract both the visual features from the input image $\varvec {{\mathcal{P}}}_I$ as well as the textual features from the associated caption. For the former, we use a state-of-the-art visual feature extraction backbone network, the ResNet architecture family while for the latter, we leverage the GLoVe word embeddings with a recurrent neural network. In addition, we also extract features that model the Theory of Reasoned Action; the *attitude of the creator* and the *social norm of the kind of post*, $\varvec {{\mathcal{P}}}$. We concatenate the three features in late fusion to make the final intent prediction. In the following sections, we describe each component in more detail.

### Intent-o-meter: approach

#### Stream 1: visual modality

The dominant modality for such social media platforms is often the visual modality, i.e., images and videos. To be consistent with prior work, we use the ResNet-18 network pretrained on the ImageNet dataset^44^ to encode the visual features^45^. We use the output of the second-to-last layer for the image representation (${\mathbb {R}}^{N \times 512}$). To fine-tune this, we then add two trainable fully-connected layers ($\phi$) with ReLU non-linearity and 0.5 dropout, to finally get $f_\textsc {visual}$.

1 $$\begin{aligned} f_\textsc {visual} = {\mathcal {S}}_1\Big (\textsc {ResNet}_{18}(\varvec {{\mathcal{P}}}_I)\Big ) \end{aligned}$$

#### Stream 2: textual modality

Prior work in multimodal learning show that visual information is often not enough to recognize human intent^46,47^. We use the user-generated captions, $\varvec {{\mathcal{P}}}_C$, of the images as a complementary cue. To encode these captions we leverage pre-trained GLoVe word embeddings^48^ to encode caption words in 50 dimensions. We use an LSTM layer, followed by two fully connected layers ($\phi$) with ReLu non-linearity and 0.5 dropout to get $f_\textsc {textual}$.

2 $$\begin{aligned} f_\textsc {textual} = {\mathcal {S}}_2\Big (\textsc {LSTM}(\textsc {GLoVe}(\varvec {{\mathcal{P}}}_C))\Big ) \end{aligned}$$

#### Stream 3: modeling TRA

As discussed previously, according to Theory of Reasoned Action (TRA), individuals who have more favorable attitudes and perceive stronger subjective norms regarding a behavior (in this case, posting particular content) are more likely to show greater intentions to execute that behavior. Many studies^39–41^ have validated the influence of TRA on users while posting content on social media, but no method exists that computationally models both these components from a post, $\varvec {{\mathcal{P}}}$. We describe this below.

#### Stream 3(a) attitude

In TRA, a user’s attitude indicates how strongly the creator believes in the post they are sharing online. Since “belief” in a post is subjective, we refer to social media psychology literature where studies have correlated engagement and frequency with social media use and in particular, one such study^49^ states, *“highly engaged youth participated on social media platforms often and in diverse ways: messaging friends, reacting to and circulating others’ posted content, and generating their own”.* We model such engagement in two ways. The first is via caption sentiments. Kruk et al.^18^ show that two different captions for the same Instagram image can completely change the overall meaning of the image-caption pair. With this intuition we compute the polarity of the sentiments expressed in the captions. We use the VADER^50^ library to compute these features.

3 $$\begin{aligned} f_\textsc {sentiment} = \textsc {VADER}(\varvec {{\mathcal{P}}}_C) \end{aligned}$$

The second way in which we model user engagement and frequency on social media is via the editing and filters applied on the images before they are posted on the various social media platforms or sophisticated cameras used for capturing images. Doing so may be reflective of the resources spent in preparing the post and indicative of the attitude the creator has towards the image they are sharing. To help our model learn this, we compute *k* image quality or visual aesthetic features, $q_1, q_2, \ldots , q_k$. These include a collection of a subset of visual aesthetic features like Auto Color Correlogram, Color and Edge Directivity Descriptor, Color Layout, Edge Histogram, Fuzzy Color and Texture Histogram, Gabor, Joint descriptor joining CEDD and FCTH in one histogram, Scalable Color, Tamura, and Local Binary Patterns extracted using the LIRE (http://www.lire-project.net/) library. As suggested by prior work, we also extract various features for color, edges, boxes and segments using Peng et al.^51^.

4 $$\begin{aligned} f_\textsc {quality} = \textsc {Image\_Quality}(\varvec {{\mathcal{P}}}_I) = \big [ q_1, q_2 \dots q_k \big ]^\top \end{aligned}$$

We concatenate the features and use fully connected layers and non-linearity to compute $f_{3_a}$.

5 $$\begin{aligned} f_\textsc {attitude} = {\mathcal {S}}_{3_a}\bigg (\big [f_\textsc {sentiment} ; f_\textsc {quality}\big ]^\top \bigg ) \end{aligned}$$

#### Stream 3(b) social norm

The goal here is to understand how well the content posted is perceived socially. The usual meaning of social norms is the set of rules that define acceptable/appropriate behaviors. However, we are trying to understand the meaning of social norm in the world of social media. One such indicator is the use of hashtags $\varvec {{\mathcal{P}}}$ with social media posts. While some creators select hashtags for their post based on relevance, but it can also be about choosing hashtags that will maximize their reach to a bigger audience. And, this is the decision that can play a huge role in the intent of the post. Furthermore, what we want to capitalize on is how social media platforms are built and are making creators select hashtags. They suggest hashtags based on whats most popular, catchy and will cause more engagement on their platform. Moreover, prior work^52,53^ has shown that hashtags are directly correlated to growing one’s social network and expanding their audience. We assume that the most influential hashtags appear first in the set of available hashtags, $\varvec {{\mathcal{P}}}_{{\mathcal {H}}} = \{ \varvec {{\mathcal{P}}}_{h_1},\varvec {{\mathcal{P}}}_{h_2}, \dots , \varvec {{\mathcal{P}}}_{h_n}\}$. This is a reasonable assumption due to the auto-suggest feature in most devices. Assuming a linear piece-wise weighting scheme, with a weight of $\frac{n-1}{n}$, for the hashtags, we use pre-trained GLoVe word embeddings^48^ to encode the words as $50-$dimensional features. We use an LSTM layer, followed by two fully connected layers with non-linearity and dropout to get $f_\textsc {social}$.

6 $$\begin{aligned} f_\textsc {social} = S_{3_b}\left( \sum _{i=1}^{n}{\frac{n - i}{n} \varvec {{\mathcal{P}}}_{h_i}}\right) \end{aligned}$$

We conclude this section by emphasizing that our current TRA model, based on caption sentiments, image aesthetics, and hashtag embeddings, is heuristic and may be one of several possible way alternatively modeling TRA. It should, accordingly, not be presumed as a gold standard way of computationally modeling TRA—that remains an open research question—and we hope this work is a stepping stone towards further research in this area.

### Fusion: inferring the perceived intent label

To fuse the four features/encodings we have computed, $f_\textsc {visual}, f_\textsc {textual}, f_\textsc {attitude}$, and $f_\textsc {social}$ from the three streams, we concatenate these features before making any individual intent inferences.

7 $$\begin{aligned} \begin{aligned} f_\textsc {concat}&= \Big [ f_\textsc {visual}, f_\textsc {textual}, f_\textsc {attitude}, f_\textsc {social}\Big ]^\top \\ f_\textsc {fuse}&= {\mathscr {S}}_\textsc {fuse}\big ( f_\textsc {concat}\big )\\ \end{aligned} \end{aligned}$$

We use two fully-connected layers followed by a softmax layer. This output is used for computing the loss and back-propagating the error back to the network.

### User study setup

**Figure 2:**
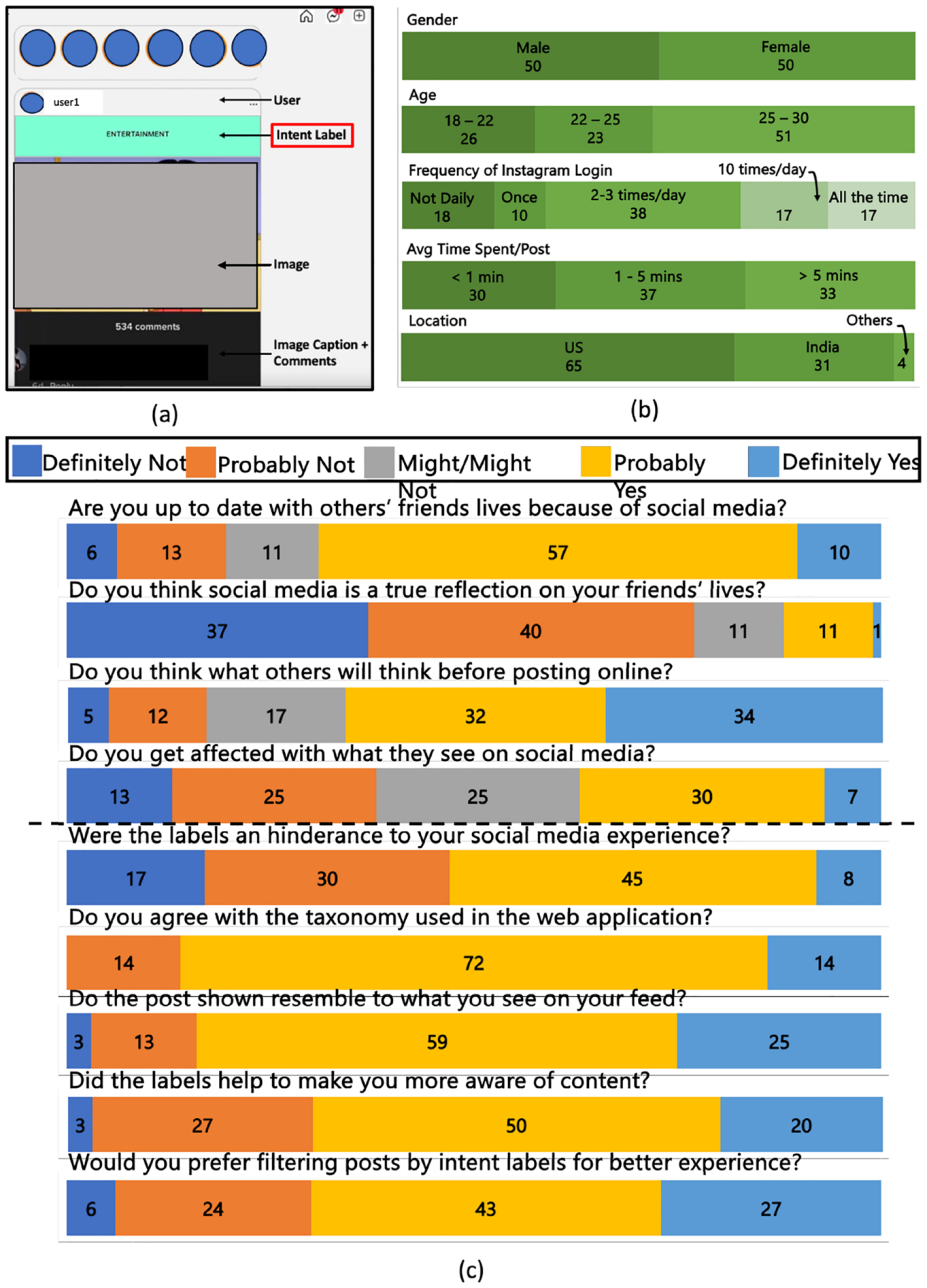
User study setup and analysis: we summarize our user study setup and findings here. In (**a**), we show a screenshot with various components highlights, in (**b**) we report the background of the 100 participants recruited for the user study and, finally in (**c**) we report the answers to the questions of the pre-questionnaire and post questionnaire.

The study consists of a web application where users interact with an “Instagram-like” interface in which the posts are taken from Intentgram. For each post, users also see an intent label for that post (highlighted in green on top in Fig. 2a). We instruct participants to scroll through the feed for 5–10 min to experience the interface.

Prior to the interacting with the interface, we ensure that (a) participants are between the ages of 18 and 30 and (b) they sign a consent form. In addition, we request them to answer a pre-study questionnaire which consists of six questions (Suppl Appendix Fig. 1a) based on their current usage of Instagram. We also provide a screen recording (https://youtu.be/9w1dj93evyA) of our web application to the users in case they have issues accessing the web application. Finally, after the task, we ask participants to answer a post-study questionnaire, that consists of another six questions to collect their feedback on our web application  (Suppl Appendix Fig. 1b).

### Ethical considerations

We note that our dataset sources Instagram posts from public profiles scraped. However, in interest of preserving privacy, we will release ResNet-18 features of all these images only. Furthermore, we provide a detailed explanation of procuring the Instagram data for reproducibility. The user study protocol was approved by the Institutional Review Boards (IRB) of the University of Maryland, College Park (IRB #1890563-2). Written informed consent was obtained from all participants and/or their legal guardian(s). The authors confirm that all methods, research, and experiments were performed in accordance with relevant IRB guidelines and regulations.

## Data

We present our perceived creator intent taxonomy and data collection procedure for Intentgram followed by a comparison with other social media intent datasets. More detailed insights are available in Suppl Appendix 1.

### Taxonomy, collection, and pre-processing

7-label taxonomy: we follow the intent taxonomy used by Kruk et al.^18^, as they also define the labels on Instagram data. We summarize this further in Table 1.

**Table 1:** Intent taxonomy: we summarize the 7-label taxonomy we adopt for Intentgram (borrowed from Kruk et al.) and the number of samples per label.

Label	# Samples	Interpretation
Advocative	9293	Advocate for a figure, idea, movement
Entertainment	8938	Entertain using art, humor, memes etc
Exhibitionist	5327	Create a self-image reflecting the person
Expressive	9800	Express emotion at an external entity
Informative	7964	Information regarding a subject or event
Promotive	4661	Promote events, products, organizations
Provocative	9289	Directly attack an individual or group
Total	55, 272	

Scraping instagram posts: we used the Apify scraper to collect Instagram posts from publicly available profiles, similar to Kruk et al.^18^. As a first step, we begin by scraping Instagram posts belonging to the seven categories (Table 1) using hashtags provided by Kruk et al. We initially collected and clustered a large number of Instagram content to understand and identify popular hashtags. Based on the frequency of usage, we choose top-10 hashtags for each of the intent labels. We have added these hashtags in Table 2 in Suppl Appendix 1.

Dataset Pre-processing: with an aim to curate a large-scale collection of publicly available Instagram posts we scrape 2000 samples for all the hashtags under consideration. Thus after the initial phase, we end up getting 1, 40, 000 posts in total. In the process of scraping content, we do not limit ourselves to users with limited number of followers, and only scrape based on the hashtags. Hence, posts scraped could be from individuals with a wide range of following including those who use social media platforms as their job. The Apify platform provides a mirror of the original Instagram posts (viable only for a short time) to download them. We then apply pre-processing and cleaning as described in Suppl Appendix “Intentgramcleaning and processing” to get the final dataset consisting of 55, 272 posts. For fair evaluation, we restrict ourselves to a total of 10, 053 samples (equally distributed across all seven categories) for the purpose of training, validation, and testing. We will release the entire dataset to facilitate further research by the community.

Dataset statistics: we also collect relevant metadata for each post such as caption, hashtags, number of likes, and number of comments. Due to privacy concerns, we release only the ResNet-18 features of the images in Instagram posts (a commonly adopted practice in social media research^18,54,55^).

### Comparing Intentgram with SOTA datasets

**Table 2:** Characteristics of intent prediction datasets: we compare Intentgram with state-of-the-art intent prediction datasets.

Datasets	Features				#Labels	Size	Source
	I	V	C	H			
MDID^18^	✓	✗	✓	✓	7	1299	Instagram
Intentonomy^17^	✓	✗	✗	^a^ ✓	28	14, 455	Unsplash
MET-Meme^21^	✓	✗	✓	✗	5	10, 045	Twitter, Weibo
							Google, Baidu
^a^Purohit et al.^36^	✗	✗	✓	✗	3	4000	Twitter
^a^MultiMET^22^	✓	✗	✓	✗	4	6109	Twitter, Facebook
MIntRec^56^	✗	✓	✓	✗	20	2224	TV series
WHYACT^35^	✗	✓	✓	✗	24	1077	YouTube videos
Intentgram	✓	✗	✓	✓	7	55, 272	Instagram

See  “Comparing INTENTGRAM with SOTA datasets” for a detailed discussion on a comparison between these datasets. I: image, V: video, C: caption, and H: hashtag.

^a^ Not available publicly.

Table 2 compares our proposed dataset, Intentgram, with state-of-the-art intent classification datasets. Intentgram uses the 7-label taxonomy (*advocative, entertainment, exhibitionist, expressive, informative, promotive, provocative*) borrowed from MDID dataset, which is based on Goffman and Hogan’s prior work^57,58^ for Instagram data. Intentgram is the most diverse in terms of available modalities and features consisting of images, captions, and hashtags. The MDID dataset^18^ also uses Instagram as the source data but is $40\times$ smaller than Intentgram. In fact, Intentgram is the largest dataset containing approximately 55K data points. Finally, we note that while the MDID, Intentonomy, MET-Mete, MultiMET and the dataset proposed by Purohit et al. are specifically intended for intent classification and social media analysis, the MIntRec and the WHYACT are in fact action prediction datasets.

## Results

Our experiments answer the following two questions: (i) Does modeling TRA result in better intent prediction in social media posts? and (ii) How does Intent-o-meter compare to state-of-the-art (SOTA) methods?

### Experimental setup

Dataset splits: we use four intent prediction datasets: Intentonomy^17^, MDID^18^, and MET-Meme^21^, and Intentgram. We used the original splits provided by the authors for Intentonomy, MDID, and MET-Meme datasets. For the purpose of experiments, we sample 10, 053 posts from Intentgram (1443, 1154, 1415, 1576, 1475, 1420, and, 1570 posts respectively for the seven intent label) and we split training, validation, and testing sets in the ratio 60 : 20 : 20, resulting in 6031, 2011, and 2011 samples for train, validation, and test sets, respectively.

Evaluation metrics: different datasets have used different metrics for evaluation. The Intentonomy dataset uses Micro F1 score and Macro F1 score. Similarly, MDID reports accuracy and AUC metric. For the MET-Meme dataset, we have reported and compared against both validation and test F1 scores. For our dataset, Intentgram we report Accuracy, AUC metric, and Micro-F1 score.

Training details: all our results were generated on an NVIDIA GeForce GTX1080 Ti GPU. Hyper-parameters for our model were tuned on the validation set to find the best configurations. We used Adam optimizer for optimizing our models with a batch size of 50. We experimented with the range of our model’s hyperparameters such as: dropout $\{0.2, 0.3, 0.4, 0.5, 0.6\}$, learning rate $\{1e^{-2}, 1e^{-3}, 1e^{-4}\}$, number of epochs $\{50, 75, 100, 125\}$, and the hidden dimension of LSTM layers $\{32, 24, 16\}$.

### Benefits of TRA in perceived intent prediction

**Table 3:** Benefit of TRA in perceived intent prediction: we highlight the importance of using TRA in addition to visual and textual features by ablating Intent-o-meter and analyzing each component in isolation.

Dataset	Metric	Experiments					
		Streams					
		1 + 2	1 + 2 + 3(a)	1 + 2 + 3(a)	1 + 2 + 3(a)	1 + 2 + 3(b)	1 + 2 + 3(a) + 3(b)
			Only VADER	Only Image Quality			Intent-o-meter
Intentonomy	F1	32.72	37.34	39.24	40.68	-	$\mathbf {40.68}$
MET-Meme	F1	38.89	45.21	43.17	47.74	-	$\mathbf {47.74}$
MDID	Acc.	54.29	55.01	54.82	55.58	57.12/55.92(*u*)	$\mathbf {58.20}$
Intentgram	Acc.	50.21	51.91	51.02	52.36	53.73/51.23(*u*)	$\mathbf {54.01}$
	AUC	73.58	75.23	74.23	76.86	75.51/74.87(*u*)	$\mathbf {79.48}$

– indicates the absence of hashtag information in the dataset. (*u*) indicates uniform weighting for hashtag embeddings. *stream* 1: visual, *stream* 2: textual, *streams* 3(a) and 3(b): TRA.

Significant values are in bold.

In Table 3, we highlight the benefit of modeling TRA, in addition to leveraging the visual and textual features obtained from images, captions, and hashtags. Specifically, we ablate Intent-o-meter on all four datasets and report the F1 score, accuracy, and the AUC. In particular, we compare the results in the first column (“$1+2$”) with the last column (“Intent-o-meter”). Our results show that leveraging TRA improves the F1 score by $7.96\%$ and $8.85\%$ on the Intentonomy and MET-Meme, results in higher accuracy by $4\%$ each on MDID and Intentgram, and increases AUC by 5.9 points on Intentgram.

We also perform additional tests where we individually analyze the individual effect of embedding the caption sentiments and image aesthetics as well as associated hashtags. In particular, the column under (“$1+2+3(a)$”) highlights the benefit of modeling caption sentiment and hashtags ordering. We also explore the impact of sentiment and hashtags, the two aspects being modeled in stream 3(a). We observe that sentiment is more helpful than image quality for all datasets except Intentonomy. This is not unexpected as it is majorly an image-based dataset. And in the (“$1+2+3(b)$”) column, we analyze Eq. 6 by comparing linear piece-wise weighting with uniform weighting with each weight set to 1, and conclude that weighting, in some form, is better. Future work involves exploring more sophisticated weighting schemes including transformer-based attention.

We believe that including and modeling TRA the way we do is incorporating human behavior to some extent and is also capturing social media characteristics (like hashtags); which probably explain the increase in the performance of Intent-o-meter. In addition to the above ablation experiment, we can also draw further evidence for TRA from our experiments comparing Intent-o-meter with state-of-the-art intent prediction methods that solely rely on visual and textual features, which we describe below.

**Table 4:** Evaluation on the MDID: we summarize the experiment results on MDID dataset here.

Method	Top-1 accuracy	AUC
Random	28.10	50.00
Gonzaga et al.^59^	54.50	84.40
Kruk et al.^18^	56.70	85.60
Intent-o-meter	$\mathbf {58.20}$	$\mathbf {89.70}$

We report top-1 accuracy and AUC score for comparisons. There are a total of seven intent labels.

Significant values are in bold.

**Table 5:** Evaluation on the Intentonomy dataset: we present experiments for intent prediction on the Intentonomy dataset.

Method	Micro F1	Macro F1
Random	7.18	6.94
Kruk et al.^18^	32.72	28.57
Jia et al.^17^	38.49	31.12
Intent-o-meter	$\mathbf {40.68}$	$\mathbf {34.71}$

We report micro F1 score and macro F1 scores for comparisons. There are a total of 28 intent labels.

Significant values are in bold.

**Table 6:** Evaluation on the MET-Meme dataset: we summarize the experiments for MET-Meme dataset here.

Method	Validation	Test
	Micro F1	
Random	23.20	22.32
Kruk et al.^18^	36.36	38.89
Xu et al.^21^	37.64	41.65
Intent-o-meter	$\mathbf {41.33}$	$\mathbf {47.74}$

We report top-1 accuracy and AUC score for comparisons. There are a total of seven intent labels.

Significant values are in bold.

**Table 7:** Evaluation on our dataset,  Intentgram **: **we summarize evaluations on Intentgram here.

Method	Top-1 accuracy	AUC	Micro F1
Random	28.10	50.00	−
Kruk et al.^18^	50.21	73.58	49.15
Intent-o-meter	$\mathbf {54.01}$	$\mathbf {79.48}$	$\mathbf {53.54}$

We report accuracy, AUC scores and micro F1 score for comparisons. There are a total of seven intent labels.

Significant values are in bold.

### Comparing Intent-o-meter with SOTA

We summarize our comparisons of our model with SOTA methods on the MDID (Table 4), Intentonomy (Table 5), MET-Meme (Table 6), and our dataset Intentgram (Table 7) respectively (not all codes provided; hence the SOTA baselines are dataset-specific).

Performance on MDID dataset: we compare against the prediction model proposed by Kruk et al.^18^(Code replicated by us due to unavailability) and Gonzaga et al.^59^. While Kruk et al. propose the use of image and captions for predicting intent labels, Gonzaga et al. create a transductive graph learning method. We observe that our model outperforms these methods by up to 3.7% in top-1 accuracy and 5.3 AUC points.

Performance on Intentonomy dataset: we compare against the prediction model proposed by Jia et al.^17^ who propose the use of hashtags as an auxiliary modality for predicting intent labels. We observe that our model outperforms their method by up to 3.59% in F1 score.

Performance on MET-Meme dataset: we compare against the baseline prediction model proposed by Xu et al.^21^ who only use image modality to predict intent labels and Kruk et al.^18^. We observe that our model outperforms these methods by up to 6.9% in F1 score.

${\text {Performance on our dataset, } \textsc {Intentgram} }$: we compare against the intent prediction model proposed by Kruk et al. We observe that our model outperforms these methods by 4% in top-1 accuracy and F1, as well as by 6 AUC points.

Conflating our results obtained from the ablation experiment in the previous section with our comparison results with SOTA methods that do not use TRA on 4 standard datasets, we find strong evidence that modeling TRA significantly improves intent prediction in terms of F1 score, top-1 accuracy, and AUC.

### Ablation experiments

**Table 8:** Choice of models for visual/textual modality: we justify the chosen models/features, ResNet-18 and GLoVe for visual and textual streams (1 and 2) respectively by comparing with some other baselines.

Dataset	Metric	Experiments					
		Stream 1			Stream 2		
		ResNet-18	ResNet-50	ResNet-101	GLoVE	Word2Vec	FastText
Intentgram	Acc.	48.31	49.75	47.12	47.19	46.53	45.91
	AUC	70.29	71.57	69.83	70.86	69.58	70.59

In Table 8, we justify the choice of our features/models for stream 1 (visual) and stream 2 (textual) in Intent-o-meter. In order to maintain consistency with prior work in social media, we employed ResNet-18 for visual features and GloVe embedding for textual features, as they are well-suited for the size of our dataset. We conducted experiments with alternative embedding models such as Word2Vec and FastText, we found that GloVe provided slightly better performance. We also tried using ResNet-50 and ResNet-101, with the former showing a marginal improvement of 1% in accuracy, while the latter resulted in decreased performance.

### Understanding human preference

Because we are inferring the perceived creator intent, it is important to understand human preferences and their reaction to these intent labels that are being displayed alongside social media posts. Towards this, we conducted a user study, similar to T-Moodifier^16^, to answer two questions: (i) do these perceived intent labels on posts make users more aware of the content they consume? and (ii) would they prefer to have their content filtered by such labels? We describe the user study setup in “Fusion: inferring the perceived intent label” and analyze the results of the study in “Understanding human preference”.

#### User study analysis

We recruit 100 participants for our user study (50 identify as female and 50 as male). We summarise statistics about the participants age and geographical locations in Fig. 2b (*rows 2,3*). We also gather information about their amount of usage of social media application, Instagram. In Fig. 2b (*row 4*), we report the frequency of social media logins and in Fig. 2b (*row 5*), we record the average time taken to publish a post by participants.

In addition to statistics about the participants, we also gather information about the role of social media in their lives. In Fig. 2c, 67% lean towards believing they are up to date with their friends lives because of social media and 77% participants also believe that social media is not a true reflection of their friends’ lives. Similarly, 37% participants report getting affected by what they see online, while 25% unsure if they are getting affected. As a testimony to our web interface, roughly half participants, 53% reported that the display of the perceived intent labels was not a hindrance to their social media application experience; 86% participants seem to in agreement with the taxonomy of intent labels used to tag posts; and 84% participants also report a resemblance to the posts shown and the posts they see on their own personal social media feeds. And finally, 70% participants reported both that the displayed intent labels helped them become aware of the content they are consuming on social media and that they would prefer filtering the content based on such intent labels.

We had also asked participants for optional suggestions, comments and feedback on the web application. A common theme among the suggestions was the presentation of the intent labels. One participant suggested color-coding intent labels; and another suggesting making intent labels optional, and letting users control if they would want to view posts with labels or without labels. Some participants appreciated the green highlighting that distinguished the labels whereas others mentioned preferring a more subtle appearance *e.g* in a corner in a smaller font. We provide a more in-depth analysis based on gender, age and social media usage in Suppl Appendix “More userstudy analysis”.

## Conclusion

We proposed Intent-o-meter, a perceived human intent prediction model for social media posts using visual and textual modalities, along with the Theory of Reasoned Action. We evaluated our model on the Intentonomy, MDID, and MET-Meme datasets. We introduced Intentgram, a dataset of 55*K* social media posts scraped from public Instagram profiles. Finally, we also developed a web application with intent labels displayed on the posts and test it with existing Instagram users.

We acknowledge that TRA may constitute one of several ways to model psychologically cognitive cues in social media posts. Using other theories that reason about human behavior like, Theory of Perceived Behavior^60^ can also be helpful for understanding human intent. We will also build upon already existing features by identifying additional features, e.g. develop better user profiling, understand a user’s social network, and their social media activity for better encapsulating a person’s motive.

Our user study indicates that tagging posts with intent labels helps users become more aware of the content consumed, and they would be open to experiment with filtering content based on the labels. However, more extensive user evaluation is required to understand how adding such perceived intent labels mitigate the negative effects of social media.

### Challenges

Social media has changed drastically over the last few years. With an increased usage of social media platforms, we do have a wealth of potential data and vast amount of insights that can be drawn from this data. However in an attempt to protect users data, platforms are increasingly limiting developer and researchers access to mining data on their platforms. We believe problem statements like inferring perceived human intent can greatly benefit if we can have access to user profile, their past posts leading up to the post we are studying and also their social network. But, we understand this is not possible. We believe that social media research in general is proposing solutions with these data restrictions and so are we. While we do believe that this makes the solutions harder, however we do not think this changes the validity of the solutions.

### Supplementary Information

Supplementary Information.

## Data Availability

We have put our dataset at https://gamma.umd.edu/researchdirections/affectivecomputing/emotionrecognition/intent_o_meter.
