# Supplementary material for: Towards determining perceived audience intent for multimodal social media posts using the theory of reasoned action
Source: Sci Rep. 2024 May 8;14:10606. doi: 10.1038/s41598-024-60299-w (PMC11078925; doi:10.1038/s41598-024-60299-w)
Supplement: Supplementary file 1 — Supplementary Information. [file 41598_2024_60299_MOESM1_ESM.pdf]

Towards Determining Perceived Audience Intent for Multimodal Social Media Posts using The Theory of Reasoned Action

Trisha Mittal[1,*], Sanjoy Chowdhury[1], Pooja Guhan[1], Snikitha Chelluri[1], and Dinesh Manocha[1,2]

[1]Department of Computer Science at the University of Maryland, College, Park, USA
[2]Department of Electrical and Computer Engineering at the University of Maryland, College, Park, USA
[*]trisha@umd.edu

1 Appendix 1

1.1 INTENTGRAM Cleaning and Processing

We list down the various hashtags used to scrape the public posts in Supplementary Table 2. Because this is a dataset scraped from Instagram, it was quite noisy and required cleaning. We mainly filter the data points in the following aspects-

1. **Duplicate Removal:** We observed that during the scraping process a lot of duplication posts got scrapped due to the modus operandi of the Apify platform. The first thing was to remove the duplicate posts.
   Another interesting observation made was many of the scraped posts had 2 or more of the hashtags under consideration thereby getting scraped more than once. For the purpose of this study, we restrict ourselves to considering posts belonging to a single category. Hence the duplicates were ignored resulting in a further reduction in sample size.
2. **Language:** In this work, we limited ourselves to posts with English captions only and therefore, discarded posts written in other languages.
3. **Non-Textual Characters:** We clean the captions of emoticons, special characters, unnecessary punctuation marks, etc.
4. **Multimodal Posts:** Since we are developing a multimodal intent prediction model, we also remove posts without hashtags and captions.

1.2 Detailed Analysis of INTENTGRAM

We summarize some insights of our dataset, INTENTGRAM in Supplementary Table 1. We observe that the number of hashtags used is more or less consistent across all the 7 categories. However, the interesting thing to note is the average number of likes in the Promotive class is significantly more than in the other classes. This might be attributed to the fact that a lot of people tend to get more influenced by such content over social media. Similarly, the average caption length is considerably higher in the Provocative class. This might be due to the fact that creators use more textual content in their posts to make their case even more strong.

1.3 Explanation of Intent Taxonomies

As mentioned in Section 2.2, there is no consensus on the intent taxonomy for social media posts. We summarize the various taxonomies that have been used for annotating social media posts with intent labels in the recent past in Supplementary Table 3. We list the various datasets and their source of social media posts too. Our decision to stick with the 7-label intent taxonomy as proposed by Kruk et al. was driven by the fact that their source of social media posts was similar to INTENTGRAM's source, Instagram.

2 Appendix 2

2.1 Building the web application interface

The aim of building this application was to make active social media users aware of an experience they could have if they had access to information about the intent behind the posts available on their social media news feed. We also wished to understand how receptive users would be to such a design. The main challenge here was to build a web application that closely resembles a platform that most participants of the study would be familiar with. Therefore, we chose to build from scratch, a web application that resembles the popular social media platform Instagram as much as possible. The UI of the web application

	Avg No. of Hashtags	Avg No. of Likes	Avg Caption Length
**Advocative**	16.71	101	198
**Entertainment**	19.29	156	151
**Exhibitionist**	16.07	53	122
**Expressive**	15.22	34	163
**Informative**	17.08	45	215
**Promotive**	14.33	331	199
**Provocative**	15.58	174	306

Supplementary Table 1. INTENTGRAM Statistics: We summarize some insights from our dataset, INTENTGRAM; average number of hashtags, average number of likes and the average length of caption for Instagram posts per Intent class label.

Intent Label	Hashtags Used to Scrape Instagram Posts
**Advocative**	#pride, #maga, #gay, #trump, #lgbt, #love, #usa, #freedom, #insta-gay, #conservative
**Entertainment**	#meme, #earthporn, #fatalframes, #earthpix, #wanderlust, #nature, #earthfocus, #naturelovers, #naturegram, #traveldiaries
**Exhibitionist**	#selfie, #ootd, #fashion, #style, #picoftheday, #beautiful, #cute, #photography, #follow, #instalike
**Expressive**	#lovehim, #merrychristmas, #christmas, #happy, #christmastree, #christmasdecor, #christmastime, #xmas, #winter, #photooftheday
**Informative**	#news, #Noticias, #hiphop, #technology, #instadaily, #podcast, #reels, #viral, #Business
**Promotive**	#ad, #NYCC22, #funkogram, #funkocollector, #Collectible, #FunkoNews, #Funkos, #Loungefly, #FPN #FunkoPOP
**Provocative**	#antifa, #redpill, #eattherich, #socialism, #antifascist, #anticapitalism, #anticapitalist, #capitalismkills, #antiracist

Supplementary Table 2. Hashtags Used for Scraping INTENTGRAM: We summarize the hashtags used to scrape Instagram posts for the 7 Intent labels.

was built entirely using React JS. For the posts, we used images, captions, and hashtags from our dataset INTENTGRAM. The web application was hosted using firebase (https://firebase.google.com). Firebase provided us with the feature of having a real-time database. It is a cloud-hosted database wherein data was stored as JSON. Any new updates made either to the data or web design were conveyed to the users instantly.

2.2 More Userstudy Analysis

User Feedback on the Web Application: We already discussed some responses in Section 5.5. Another suggestion was to provide a feedback mechanism for users to report an incorrect intent label, and one participant suggested extending this to a multi-label classification as some posts seemed relevant for multiple labels.

More in-depth user study analysis: We filtered user study responses by age, gender, location, frequency of use, and frequency of posting (Figures 2-6). Although most trends seem consistent across the various parameters, we make some interesting observations. First, the percentage of people open to filtering social media content by intent labels increases as people grow older. The question asking if users are affected by social media revealed that females are more affected by other users' posts than men. Perhaps the most interesting observation is that the same percentage of participants worry about what others will think in each posting frequency category.

Dataset	# Intent labels	Labels
**MDID**[?]	7	advocative, entertainment, exhibitionist, expressive, informative, promotive, provocative
**MET-Meme**[?]	5	entertaining, expressive, interactive, offensive, other
**Purohit et al.**[?]	3	seeking, offering, none
**MultiMET**[?]	4	persuasive, descriptive, expressive, others
**NYT Survey**[?]	5	entertaining, self-fulfillment, promotive, grow relationships, define ourselves
**Intentonomy**[?]	28	Attractive, BeatCompete, Communicative, CreativeUnique, CuriousAdventurousExcitingLife, EasyLife, EnjoyLife, FineDesignLearnArt-Arch, FineDesignLearnArt-Art, FineDesignLearnArt-Culture, GoodParentEmocloseChild, Happy, HardWorking, Harmony, Health, InLove, InLoveAnimal, InspirOthers, ManagableMakePlan, NatBeauty, PassionAbSmthing, Playful, ShareFeelings, SocialLifeFriendship, SuccInOccupHavGdJob, TeachOthers, ThngsInOrdr, WorkILike
**INTENTGRAM**	7	advocative, entertainment, exhibitionist, expressive, informative, promotive, provocative

Supplementary Table 3. **Social Media Intent Taxonomies:** We summarize the various social media intent taxonomies proposed in the recent past.

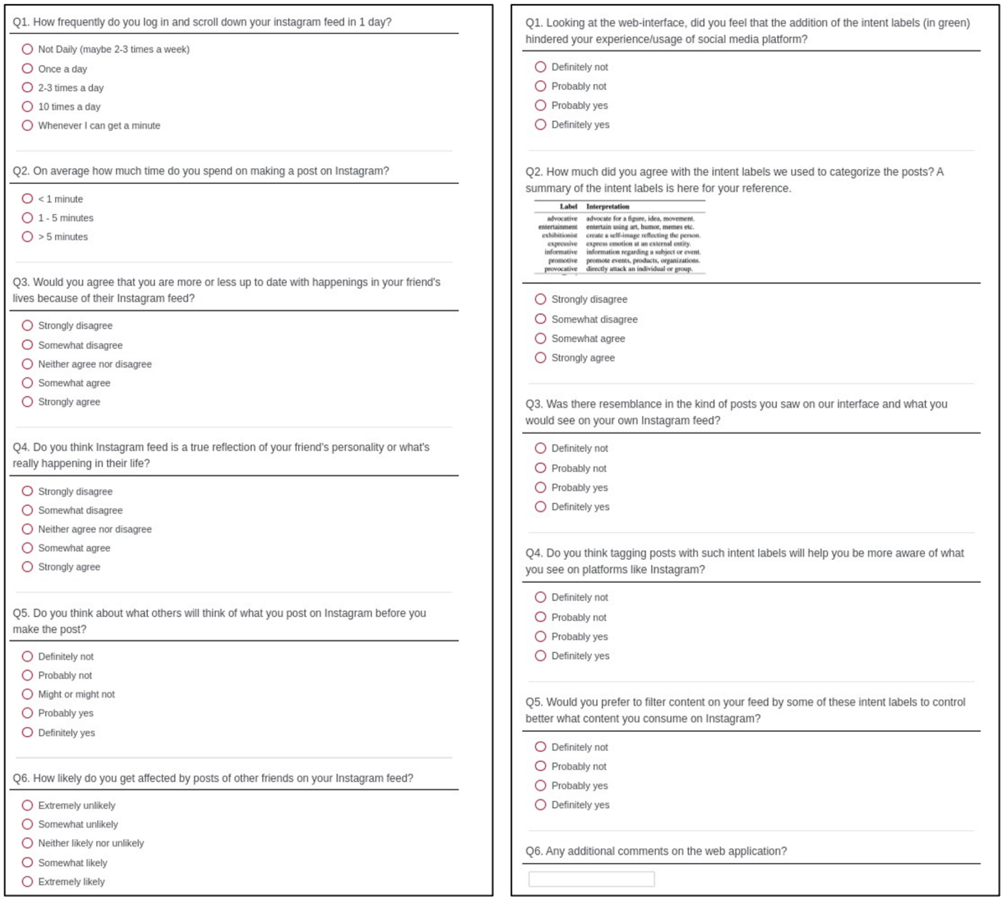

Q1. How frequently do you log in and scroll down your instagram feed in 1 day?
- Not Daily (maybe 2-3 times a week)
- Once a day
- 2-3 times a day
- 10 times a day
- Whenever I can get a minute

Q2. On average how much time do you spend on making a post on Instagram?
- < 1 minute
- 1 - 5 minutes
- > 5 minutes

Q3. Would you agree that you are more or less up to date with happenings in your friend's lives because of their Instagram feed?
- Strongly disagree
- Somewhat disagree
- Neither agree nor disagree
- Somewhat agree
- Strongly agree

Q4. Do you think Instagram feed is a true reflection of your friend's personality or what's really happening in their life?
- Strongly disagree
- Somewhat disagree
- Neither agree nor disagree
- Somewhat agree
- Strongly agree

Q5. Do you think about what others will think of what you post on Instagram before you make the post?
- Definitely not
- Probably not
- Might or might not
- Probably yes
- Definitely yes

Q6. How likely do you get affected by posts of other friends on your Instagram feed?
- Extremely unlikely
- Somewhat unlikely
- Neither likely nor unlikely
- Somewhat likely
- Extremely likely

Q1. Looking at the web-interface, did you feel that the addition of the intent labels (in green) hindered your experience/usage of social media platform?
- Definitely not
- Probably not
- Probably yes
- Definitely yes

Q2. How much did you agree with the intent labels we used to categorize the posts? A summary of the intent labels is here for your reference.

Label	Interpretation
advocative	advocate for a figure, idea, movement.
entertainment	entertain using art, humor, memes etc.
exhibitionist	create a self-image reflecting the person.
expressive	express emotion at an external entity.
informative	information regarding a subject or event.
promotive	promote events, products, organizations.
provocative	directly attack an individual or group.

- Strongly disagree
- Somewhat disagree
- Somewhat agree
- Strongly agree

Q3. Was there resemblance in the kind of posts you saw on our interface and what you would see on your own Instagram feed?
- Definitely not
- Probably not
- Probably yes
- Definitely yes

Q4. Do you think tagging posts with such intent labels will help you be more aware of what you see on platforms like Instagram?
- Definitely not
- Probably not
- Probably yes
- Definitely yes

Q5. Would you prefer to filter content on your feed by some of these intent labels to control better what content you consume on Instagram?
- Definitely not
- Probably not
- Probably yes
- Definitely yes

Q6. Any additional comments on the web application?

(a) (b)

Supplementary Figure 1. **UserStudy Questionnaires** : We show a screenshot of the two questinnaires used as a part of the userstudy. In (a), we show the 6 questions participants are asked before they see the web interface. In (b), we show a screenshot of the 6 questions participants are asked after they see the web interface.

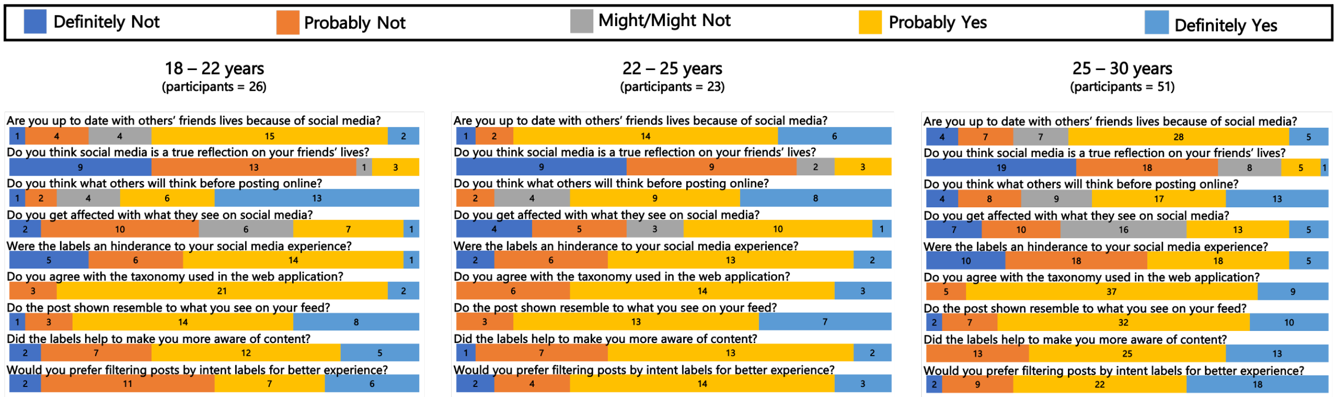

Supplementary Figure 2. User study responses by age

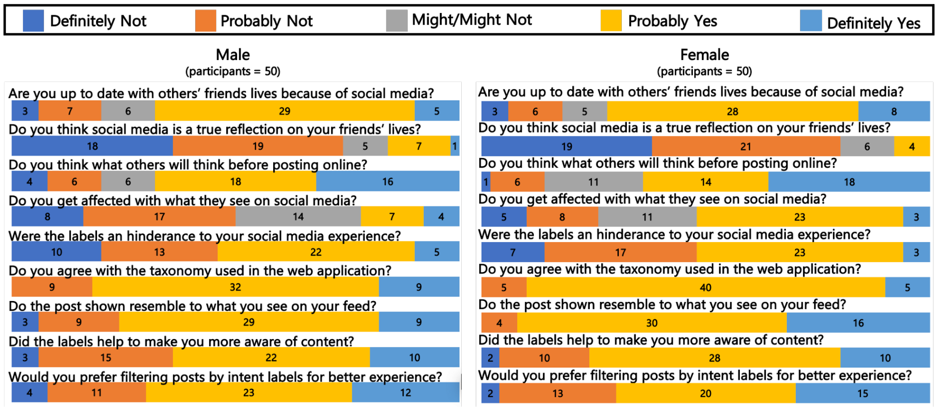

Supplementary Figure 3. User study responses by gender

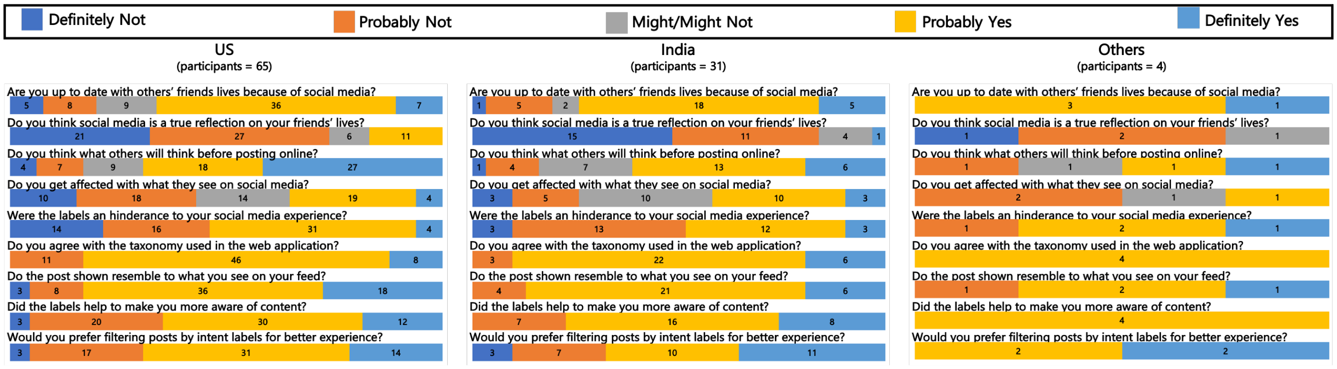

Supplementary Figure 4. User study responses by location

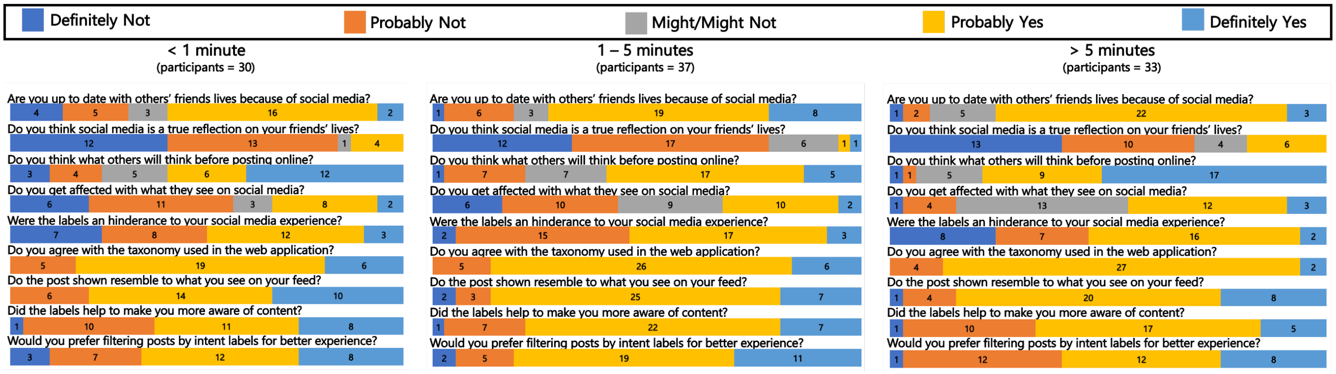

Supplementary Figure 5. User study responses by frequency of use

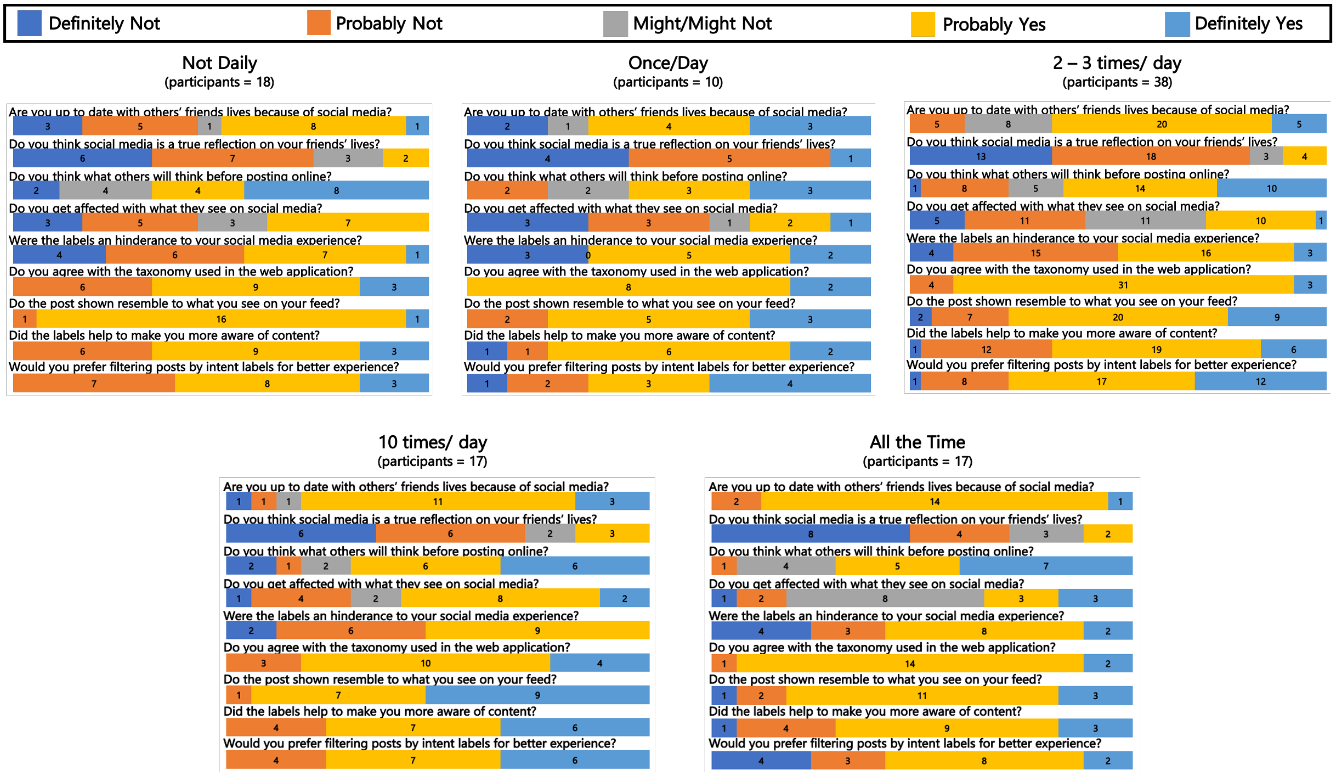

Supplementary Figure 6. User study responses by post frequency
